# Angiomes plans et laser à colorant pulsé: étude des facteurs pronostiques dans une série marocaine de 74 cas

**DOI:** 10.11604/pamj.2016.25.218.9229

**Published:** 2016-12-06

**Authors:** Bouchra Baghad, Soumiya Chiheb, Hakima Benchikhi

**Affiliations:** 1 Université Hassan II, Hôpital Ibn Rochd, Casablanca, Maroc

**Keywords:** Angiomes plans, laser à colorant pulsé, facteurs pronostiques, Port wine stains, pulsed dye laser, prognostic factors

## Abstract

Le laser à colorant pulsé (LCP) est actuellement le traitement de référence des Angiomes Plans (AP). Cependant, les critères cliniques prédictifs d'une bonne ou d'une mauvaise réponse ne sont pas encore clairement établis dans notre contexte. Notre but était de déterminer les facteurs de mauvaise /bonne réponse des AP traités par le LCP dans la population marocaine. Une étude rétrospective des patients suivis pour AP a été réalisée au service de dermatologie du CHU Ibn Rochd de Casablanca entre Janvier 2008 et Décembre 2013. Nous avons collecté les paramètres cliniques suivants : âge, sexe, localisation, antécédents, paramètres utilisés, nombre de séances, phototype et la satisfaction des médecins soignants basée sur le blanchiment des lésions. La bonne réponse a été définie par le palissement de 50% des lésions au bout de la 6ème séance. Les patients ont été contactés par téléphone afin d'évaluer leur degré de satisfaction. Ces résultats ont été corrélés aux paramètres cliniques sus cités. Un seuil de signification de 0.05 a été utilisé. Soixante-quatorze patients ont été éligibles. Le sexe féminin représentait 69% et l'âge médian était de 18 ans. La localisation au visage était prédominante (94%). L'étude comparative des groupes bons / mauvais répondeurs a montré que l'âge moyen du groupe bon répondeur était inférieur au groupe mauvais répondeur avec une différence significative (p = 0.047). Le nombre de séances du groupe bon répondeur était plus élevé (p = 0.044). Les paramètres étaient variables d'un patient à un autre. Aucune différence n'a été notée concernant le type de peau entre les deux groupes. La localisation la mieux blanchie était la zone V2. Cette étude a montré que les AP pris en charge à un âge jeune en plusieurs séances avaient une réponse thérapeutique supérieure dans notre contexte. Ceci implique l'intérêt du diagnostic précoce et une prise en charge rapide à des intervalles courts afin d'améliorer les résultats et de minimiser les effets indésirables.

## Abstract (en)

Pulsed dye laser (LCP) is currently the gold standard for Port wine stains (PWS) treatment. However, predictive clinical criteria indicating a right or poor response are not yet clear in our context. This study aims to determine the factors associated with poor/good response in Moroccan patients with PWS treated with LCP. We conducted a retrospective study of patients treated for PWS at the dermatology department at the CHU Ibn Rochd in Casablanca between January 2008 and December 2013. We collected the following clinical parameters: age, sex, location, history, parameters used, number of sessions, phototype and physician satisfaction with outcome of the bleaching therapy. A good response was defined by the achievement of 50% lesional lightening at the end of the 6th session. Patients were contacted by telephone to measure their satisfaction. These results were correlated with the clinical parameters mentioned above. We set our significance level at 0.05. Seventy-four patients were eligible The female sex represented 69% and the median age was 18 years. It occurred predominantly on the face (94%). The comparative study of good/poor responders showed that the mean age in the group of good responders was lower than that of poor responders with a significant difference (p = 0.047). The number of sessions in the group of good responders was higher (p = 0.044). The parameters were variable from one patient to another. There was no difference in the type of skin between the two groups. The best bleached location was the area V2. This study showed that patients undergoing several PWS treatment sessions at a young age had a superior therapeutic response in our context. This highlights the role of early diagnosis and short interval management to improve outcomes and minimize adverse effects.

## Introduction

Les angiomes plans (AP) appartiennent au groupe des malformations vasculaires congénitales d'après la classification de l'International Society for the Study of Vascular Anomalies (ISSVA) [1]. Les AP surviennent chez 0.3% des nouveaux nés [2]. Les AP entraînent de graves perturbations sur le plan psychologique et social en raison de leur localisation affichante: 90% des AP se situent sur l'extrémité céphalique et le cou [3]. Ainsi le traitement apparaît essentiel. Le laser à colorant pulsé (LCP) est le traitement de référence de l'AP [4]: il répond au principe de la photothermolyse sélective. Cependant, de multiples facteurs cliniques et physiques limitent l'efficacité du LCP. En effet, les vaisseaux de l'AP sont hétérogènes : seuls les vaisseaux d'une certaine profondeur et d'une certaine taille seront détruits. En conséquent, cela induirait une augmentation du nombre de séances sans amélioration avec le risque d'apparition des complications cutanées, dont les principales sont les complications pigmentaires (hyperpigmentation, achromies), les cicatrices atrophiques ou hypertrophiques, les croûtes et les bulles épidermiques [5]. A l'heure actuelle, les critères cliniques prédictifs d'une bonne ou d'une mauvaise réponse ne sont pas encore clairement établis particulièrement chez la population maghrébine. Cette étude a pour but de déterminer et d'évaluer les facteurs pronostiques de réponse thérapeutique des AP traités par LCP à travers une série de patients suivis au service de Dermatologie de l'hôpital Ibn Rochd Casablanca.

## Méthodes

**Patients:** les patients ayant un AP et traités par LCP au CHU Ibn Rochd de Casablanca ont été inclus dans cette étude de janvier 2008 à décembre 2013. Nous avons retenu toutes les localisations ainsi que les AP rentrant dans le cadre d'un groupement syndromique. Chaque patient avait bénéficié d'une consultation pré-thérapeutique ayant pour objectif de l'informer sur la procédure du traitement. Conduite du traitement : Les patients ont été traités par un LCP (Candela^®^), longueur d'onde : 595 nm. Chez les enfants, une anesthésie locale était utilisée sur les zones à traiter (crème EMLA^®^). L'intervalle entre les séances était d'une séance / 2 mois.

**Méthodes d'évaluation de l'efficacité du LCP:** une fiche d'information a été utilisée afin de réunir les facteurs cliniques suivants: Age - Sexe- Antécédents-Localisation- Paramètres utilisés - Nombre de séances- Phototype - Effets indésirables. L'évaluation de la réponse clinique par le médecin traitant était basée sur le pâlissement des lésions. Le seuil de bonne /mauvaise réponse clinique était défini par un pâlissement de 50% de l'AP au bout de 6 séances. Les patients ont été contactés par téléphone afin d'apprécier leur satisfaction après le traitement par LCP selon l'échelle suivante: aucune amélioration (0%) - Faible amélioration (1 à 25%) - Légère amélioration (26 à 50%) - Bonne amélioration (51 à 75%) - Eclaircissement quasi-total (76 à 100%). Une comparaison entre les bons/mauvais répondeurs a été réalisée afin de mettre en évidence les facteurs de réponse thérapeutique.

**Outils statistiques:** le logiciel SPSS (version 20) a été utilisé pour l'analyse des valeurs paramétriques et pour détecter les différences statistiques entre les groupes bon/mauvais répondeurs au laser avec un seuil significatif de 0.05.

## Résultats

Nous avons colligé 74 patients entre 2008 et 2013 (51 femmes et 23 hommes). Le Tableau 1 représente les principales caractéristiques épidémiologiques de nos patients. Le sexe féminin représentait 69% des patients avec une médiane de 18 ans (2-44). La localisation au visage représentait 91% alors que les autres localisations (extrémités, corps) représentaient 6%. La localisation faciale la plus fréquente était la V2 (29, 7%). Les AP rentrant dans le cadre d'un groupement syndromique étaient les suivants : Syndrome de Sturge Weber Krabbe: 3 cas; Syndrome de Klippel Trenauney: un cas.

**Tableau 1:** Profil épidémiologique des patients suivis pour angiomes plans à l’Hôpital Ibn Rochd entre 2008-2013

Caractéristiques	Résultats
**Sexe, n (%)**	
Masculin	23 (31)
Féminin	51 (69)
Age médian (ans)	18 (2-44)
**Localisation, n (%)**	
Face	68 (91.9)
V1	4
V2	22 (29,7)
V1V2	7
V3	7
Lèvres	5
Inter sourcilières	4
Nez	4
Autres	6 (8.1)

**Figure 1:**
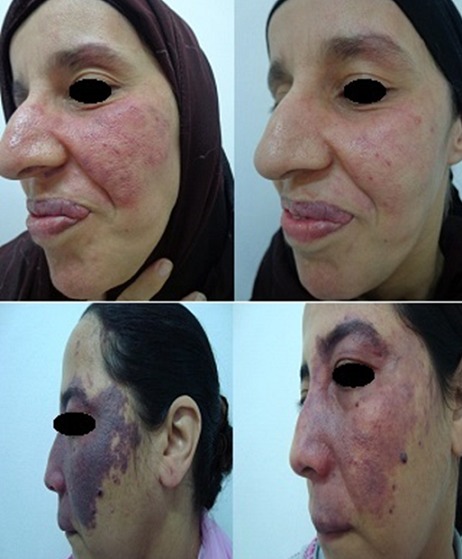
Exemple de bonne réponse de deux patients traités par Laser à colorant Pulsé

**Figure 2:**
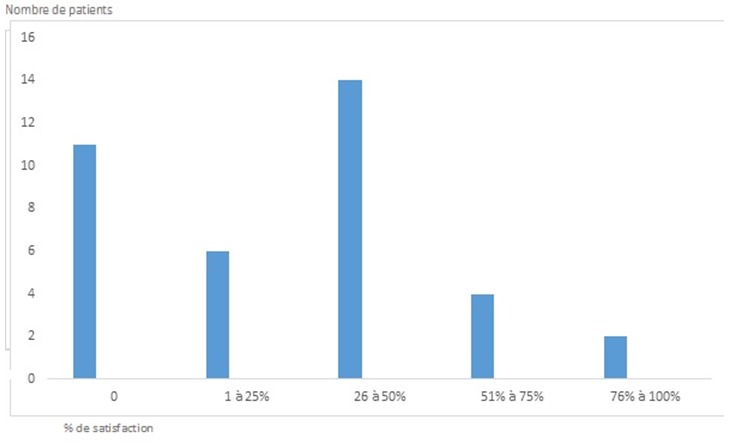
Echelle de satisfaction de 37 patients ayant un angiome plan traité par LCP suivis à l’hôpital Ibn Rochd entre 2008 et 2013

Le groupe bon répondeur (65%) avait un âge médian inférieur (19 vs 21) à celui du mauvais répondeur (35%) avec une différence statistiquement significative (p = 0.047). Aussi, le groupe bon répondeur avait en moyenne un nombre de séances de 10 séances comparé à 7 pour le mauvais répondeur avec une différence significative (p = 0.044). Le phototype III était prédominant dans les deux groupes (Tableau 2). Le LCP a été toléré de manière équivalente entre les deux groupes, les principaux effets rapportés étaient: l'érythème, l'œdème et le purpura (Figure 1). Les appréciations des patients collectés par téléphone basées sur l'éclaircissement de l'AP ont recueilli 37 réponses. La majorité des patients (38%) était dans l'intervalle de satisfaction entre 25% et 50%. La Figure 2 décrit la satisfaction des patients.

**Tableau 2:** Particularités cliniques des 74 patients porteurs d’angiomes plans (appréciation des médecins) suivis à l’Hôpital Ibn Rochd entre 2008-2013

	Bon répondeurs n(48)	Mauvais répondeurs n(26)	p
-Age médian, année	19(2-39)	21 (12-40)	0.047
-Sexe F vs M	30 vs 18	21 vs 5	0.224
-Nombre de séances (moyenne)	10	7	0.044
-Phototype			
3	81%	75%	0.925
4	15%	24%	
2	4%	1%	
-Localisation			
VI	5%	7.5%	0.272
VII	60%	35%	
VIVII	25%	18%	
VIII	8 %	15%	
Autres	2 %	24.5%	
-Pièce à main (mm)			
10	70%	50%	0.238
7	30%	50%	
-Fluence	8 à 12	8 à 11	0.130
-Temps d’exposition (ms)	1.5 à 3	1.5 à 3	0.904

## Discussion

Notre étude a permis de montrer que les facteurs prédictifs de bonne réponse au laser LCP des AP dans notre contexte étaient l'âge précoce du début de traitement et le nombre élevé de séances de LCP. Il faut dire que notre étude était rétrospective et basée sur des dossiers de patients non consécutivement suivis et que le blanchiment des AP a été déterminé par des facteurs qualitatifs : photographies, appréciation des médecins et des patients. Cela dit, notre étude rapporte des renseignements cliniques spécifiques de la population marocaine avec des paramètres adaptés aux types de peau prédominants dans ce contexte. Des mesures quantitatives de la couleur de la lésion auraient été souhaitables pour compléter l'étude. Bien que le LCP soit le traitement de référence des AP, la plupart des études au cours des deux dernières décennies ont montré qu'uniquement 20% des AP pouvaient blanchir complètement avec le LCP et que 20% à 30% semblaient ne pas répondre [6].

Les bons répondeurs étaient âgés en majorité de moins de 20 ans dans notre série : le plus jeune était un enfant de 2 ans montrant un retard diagnostique. Ceci peut être expliqué par le manque de sensibilisation et de connaissance de la population marocaine de cette pathologie et de ses opportunités thérapeutiques. L'étude de Anolik et al [7, 8] sur 49 patients âgés de moins de 6 mois traités par LCP à 4 et 6 semaines d'intervalle a montré un blanchiment moyen de 88.6% sans aucun effet néfaste à long terme. Ainsi l'âge jeune est considéré le facteur majeur de bonne réponse au LCP. La principale localisation était le visage, expliquée par l'aspect inesthétique gênant, poussant les patients à consulter et à bénéficier de séances de Laser. La localisation la mieux blanchie dans cette série était la V2. Dans son étude, Renfro et al ont trouvé que les lésions localisées dans la région péri orbitale, la partie latérale des joues, le tronc, la partie proximale du bras avaient le plus grand taux de palissement. Les zones malaires et les membres distaux répondaient moins bien [9, 10].

Dans notre étude, le phototype III était prédominant chez tous les patients expliqué par l'ethnie marocaine. Ceci, ne permet pas de discuter avec précision les différents phototypes et leurs réponses au LCP. Les personnes à peau noire ne sont pas connues bons répondeurs au Laser en raison de la plus grande concentration en mélanine qui rentre en compétition avec l'oxyhémoglobine. Ainsi, les phototypes Fitzpatrick I, II et III sont corrélés à une meilleure réponse au LCP [11]. Les femmes ont représenté la population la plus concernée dans cette étude ce qui suggère que le sexe féminin est un facteur de bon pronostic. Ceci peut être expliqué par l'assiduité et l'intérêt pressant des femmes à soigner l'aspect esthétique du visage. Nos paramètres utilisés étaient variables. Les meilleurs résultats étaient : un spot de 7mm (p = 0.9), nombre de joules de 10 (p = 0.13) et une durée de pulsation de 3 ms (p = 0.23). Cette variété est expliquée par le changement des paramètres au cours du traitement suivant l'évolution clinique et les effets secondaires rencontrés.

En effet, les paramètres de laser tel que la durée de pulsation et le diamètre du spot peuvent être modifiés pour améliorer les résultats des AP traités par LCP particulièrement pour les formes résistantes à une durée d´impulsion particulière ou pour les AP qui sont composées de vaisseaux de différent calibre [12–15]. Il est important de noter que des fluences élevées peuvent être nécessaires lors de l´utilisation d'une durée de pulsation plus longue [16]. L´augmentation de la taille du spot permettrait également une meilleure pénétration en profondeur et en largeur des structures vasculaires assurant une meilleure efficacité du laser [17]. Dans le LCP, le diamètre des spots varie de 7 à 10 mm.

Bien que notre étude ait utilisée un intervalle identique entre les séances de LCP, de nombreuses études démontrent l'intérêt majeur d'un traitement par LCP à un rythme rapproché particulièrement pour les enfants [9]. Ceci renforce la probabilité d´effacement de l'AP avant la petite enfance permettant ainsi un impact psychologique moindre. Cela dit, les études ayant analysé les différents intervalles du traitement dans la gestion de l'AP avec le LCP restent peu nombreuses. Une enquête auprès des 45 membres du British Skin Laser Study group a révélé que 84% des répondants considéraient que 2 à 3 mois était l´intervalle optimal pour le traitement par LCP [18, 19].

Ainsi, un traitement à des intervalles rapprochés serait également un facteur de bon pronostic des AP traités par LCP. La dermoscopie [20] est un outil qui pourrait être utilisé afin de définir la dose minimale et efficace lors de la première séance de LCP. Une étude japonaise avait pour objectif d'évaluer les résultats dermoscopiques avant et après une séance de LCP sur 6 patients suivis pour AP pour une durée de trois mois. Cette étude a démontré que la dose de fluence minimale efficace peut être prédite en observant les changements dermoscopiques immédiats après la séance de laser permettant ainsi au praticien de prédire la dose efficace et prévenir les effets secondaires cutanés. Ainsi cet outil pourrait aider à prédire la bonne réponse ou non des AP au LCP.

## Conclusion

En conclusion, les AP pris en charge à un âge jeune avec un nombre de séances élevé avaient une réponse clinique statistiquement supérieure dans cette série. Ceci implique l'intérêt du diagnostic précoce et une prise en charge rapide à des intervalles courts afin d'améliorer les résultats et de minimiser les effets indésirables. Les autres facteurs tels le phototype et la localisation n'ont pas été impliqués comme facteurs influençant l'évolution des AP. Plusieurs approches prometteuses à développer peuvent prédire l'efficacité thérapeutique telle l'exploration dermoscopique. Concernant les AP résistants au LCP d'autres alternatives thérapeutiques sont à proposer comme le laser Nd-Yag.

### Etat des connaissances actuelles sur le sujet

- Angiomes plans: malformations vasculaires congénitales à retentissement esthétique majeur;
- Le laser à colorant pulsé est le traitement de référence;
- La réponse thérapeutique au laser vasculaire est variable.

### Contribution de notre étude à la connaissance

- La réponse est meilleure chez les sujets jeunes ayant un nombre élevé de séances quel que soit le phototype;
- La localisation qui répond le mieux est la V2 au visage;
- Le phototype foncé répond moins bien que le clair.
